# Poly-arginine-18 (R18) Confers Neuroprotection through Glutamate Receptor Modulation, Intracellular Calcium Reduction, and Preservation of Mitochondrial Function

**DOI:** 10.3390/molecules25132977

**Published:** 2020-06-29

**Authors:** Gabriella MacDougall, Ryan S. Anderton, Amy Trimble, Frank L. Mastaglia, Neville W. Knuckey, Bruno P. Meloni

**Affiliations:** 1 Perron Institute for Neurological and Translational Science, Nedlands, WA 6009, Australia; ryan.anderton@nd.edu.au (R.S.A.); amy.trimble1@my.nd.edu.au (A.T.); francis.mastaglia@perron.uwa.edu.au (F.L.M.); neville.knuckey@health.wa.gov.au (N.W.K.); bruno.meloni@perron.uwa.edu.au (B.P.M.); 2 Institute for Health Research, School of Heath Sciences and Institute for Health Research, The University Notre Dame, Fremantle, WA 6160, Australia; 3 Centre for Neuromuscular and Neurological Disorders, The University of Western Australia, Perth, WA 6009, Australia; 4 Department of Neurosurgery, Sir Charles Gairdner Hospital, QEII Medical Centre, Nedlands, WA 6008, Australia

**Keywords:** poly-arginine-18 (R18), cationic arginine-rich peptides (CARPs), neuroprotection, ROS, mitochondrial membrane potential (ΔΨm), ionotropic glutamate receptors

## Abstract

Recent studies have highlighted that a novel class of neuroprotective peptide, known as cationic arginine-rich peptides (CARPs), have intrinsic neuroprotective properties and are particularly effective anti-excitotoxic agents. As such, the present study investigated the mechanisms underlying the anti-excitotoxic properties of CARPs, using poly-arginine-18 (R18; 18-mer of arginine) as a representative peptide. Cortical neuronal cultures subjected to glutamic acid excitotoxicity were used to assess the effects of R18 on ionotropic glutamate receptor (iGluR)-mediated intracellular calcium influx, and its ability to reduce neuronal injury from raised intracellular calcium levels after inhibition of endoplasmic reticulum calcium uptake by thapsigargin. The results indicate that R18 significantly reduces calcium influx by suppressing iGluR overactivation, and results in preservation of mitochondrial membrane potential (ΔΨm) and ATP production, and reduced ROS generation. R18 also protected cortical neurons against thapsigargin-induced neurotoxicity, which indicates that the peptide helps maintain neuronal survival when intracellular calcium levels are elevated. Taken together, these findings provide important insight into the mechanisms of action of R18, supporting its potential application as a neuroprotective therapeutic for acute and chronic neurological disorders.

## 1. Introduction

Glutamate excitotoxicity is a critical neurodamaging event responsible for neuronal death in acute forms of brain injury, such as stroke, traumatic brain injury (TBI) and hypoxic-ischaemic encephalopathy (HIE), as well as chronic neurodegenerative disorders such as Alzheimer’s disease (AD) [1,2], Huntington’s disease (HD) [3,4], Parkinson’s disease (PD) [5,6], and amyotrophic lateral sclerosis (ALS) [7,8]. The excessive release of the excitatory neurotransmitter glutamate from pre-synaptic neurons can trigger the overactivation of ionotropic glutamate receptors (iGluRs), such as N-methyl-D-aspartate (NMDAR), α-amino-3-hydroxy-5-methyl-4-isoxazolepropionic acid (AMPAR), and kainic acid (KAR) receptors [9]. The overactivation of iGluRs causes excessive neuronal calcium uptake and triggers a range of harmful intracellular biochemical cascades, culminating in cell death [9]. As such, the modulation of intracellular calcium concentrations and the preservation of intracellular organelles that store calcium, primarily the mitochondria, are critical neuroprotective targets against excitotoxic neuronal injury [10].

Mitochondria represent an important regulator of intracellular calcium levels, due to several direct and indirect mechanisms. For example, mitochondria can sequester calcium via the mitochondrial calcium uniporter (MCU), cause calcium release from internal stores (i.e., endoplasmic reticulum; ER), and supply ATP for plasma membrane calcium extrusion pumps [see reviews [11,12,13]. However, excessive intracellular calcium levels can cause a toxic influx of calcium into mitochondria, loss of the mitochondrial membrane potential (ΔΨm) across the inner mitochondrial membrane, and inhibition of the electron transport chain (ETC) and oxidative phosphorylation, leading to reduced ATP synthesis. In addition, uncoupling of the ETC leads to increased generation of reactive oxygen species (ROS), further contributing to mitochondrial dysfunction and the release of pro-apoptotic factors, such as cytochrome c and apoptosis-inducing factor (AIF), and ultimately the demise of the cell. While several key cell death pathways are involved in neuronal NMDA and non-NMDA receptor-mediated excitotoxicity, mitochondrial dysfunction is considered a critical event [12,13,14,15,16,17].

Recent studies in our laboratory have identified cationic arginine-rich peptides (CARPs), which include poly-arginine peptides, have intrinsic neuroprotective and cell-penetrating properties (reviewed in [18]). In particular, we have demonstrated that poly-arginine-18 (R18, 18-mer of arginine) is neuroprotective in in vitro neuronal excitotoxicity models and in in vivo rodent and non-human primate models of stroke [19,20,21,22,23,24], as well as rodent models of HIE [25,26] and TBI [27,28]. Given the neuroprotective properties of R18, it is imperative that the molecular pathways that underlie its neuroprotective action are fully elucidated in order to gauge its therapeutic potential. We have previously demonstrated that R18 can reduce glutamic acid-induced excitotoxic neuronal death and intracellular calcium influx, and that the poly-arginine-12 peptide (R12) reduces cell surface neuronal NMDA receptor levels [29]. Because of their cell-penetrating properties, CARPs have additional intracellular mechanisms of action, including beneficial effects on mitochondrial function and structural integrity [30]. However, these have not been fully investigated.

In this study, we aimed to confirm that the anti-excitotoxic properties of the R18 peptide were also associated with the preservation of mitochondrial function in cortical neurons. We explored the ability of R18 to attenuate excessive calcium influx and excitotoxic neuronal death induced by a variety of ionotropic glutamate receptor agonists, namely glutamate, NMDA, KA, and AMPA. Given that mitochondria are a critical modulator of intracellular calcium levels during neuronal excitotoxicity and are responsible for a significant share of the ensuing oxidative stress and energy collapse, we examined whether R18 could preserve mitochondrial bioenergetics, in particular via the maintenance of the mitochondrial membrane potential (ΔΨm) and ATP production, and through limiting ROS generation. Lastly, we investigated whether R18 could prevent neurotoxicity following thapsigargin-mediated inhibition of ER calcium uptake, representing a receptor-independent mechanism for raising intracellular calcium levels.

## 2. Results

### 2.1. R18 is Neuroprotective against Different Ionotropic Glutamate Receptor Agonists

**Figure 1:**
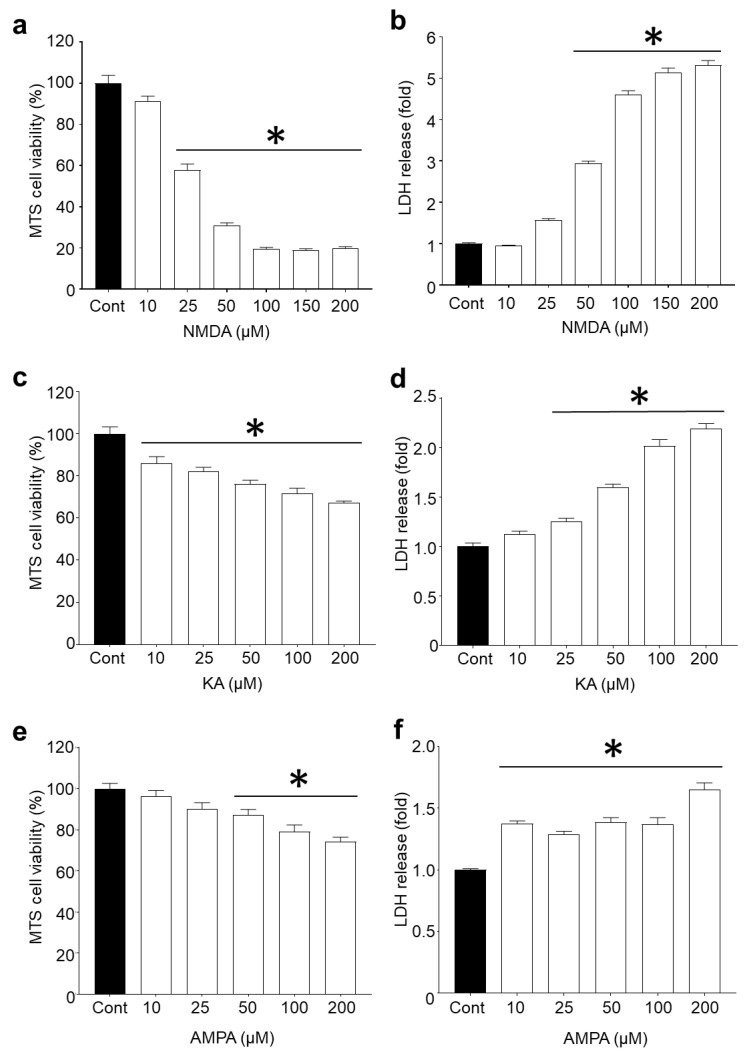
Ionotropic glutamate receptor agonists induce dose-dependent neuronal death at 24 h post-exposure. Dose–response studies of DIV10 primary cortical neurons exposed to ionotropic glutamate receptor agonists (10–200 µM) for 5 min. Neuronal viability and cell death were measured using MTS and LDH assays, respectively, at 24 h post-exposure to (**a**,**b**) NMDA, (**c**,**d**) KA, and (**e**,**f**) AMPA. Absorbance values were adjusted to represent cell viability (untreated control as 100%) and fold change in LDH release. Values are means ± SEM; *n* = 3, with a minimum of eight biological replicates per sample in each experiment; * *p* < 0.05. Experiments were conducted at least twice with independent neuronal cultures.

Since L-glutamic acid can activate NMDA, AMPA and KA ionotropic receptors, the involvement of each glutamate receptor agonist in excitotoxicity and the potential capacity of R18 to reduce toxicity was examined. Dose–response studies revealed that cortical neurons were most sensitive to NMDA, followed by KA and AMPA receptor overstimulation (Figure 1). Based on CellTiter 96^®^ AQueous Non-Radioactive Cell Proliferation MTS assay (MTS) and CytoTox 96^®^ Non-Radioactive Cytotoxicity LDH release (LDH) assays, exposure of cortical neurons to NMDA at concentrations of ≥100 µM resulted in > 90% cell death (*p* < 0.0001). In contrast, exposure of cortical neurons to KA and AMPA at 200 µM (highest concentration examined) resulted in approximately 30% (*p* < 0.0001) and 20% (*p* < 0.0001) cell death, respectively.

**Figure 2:**
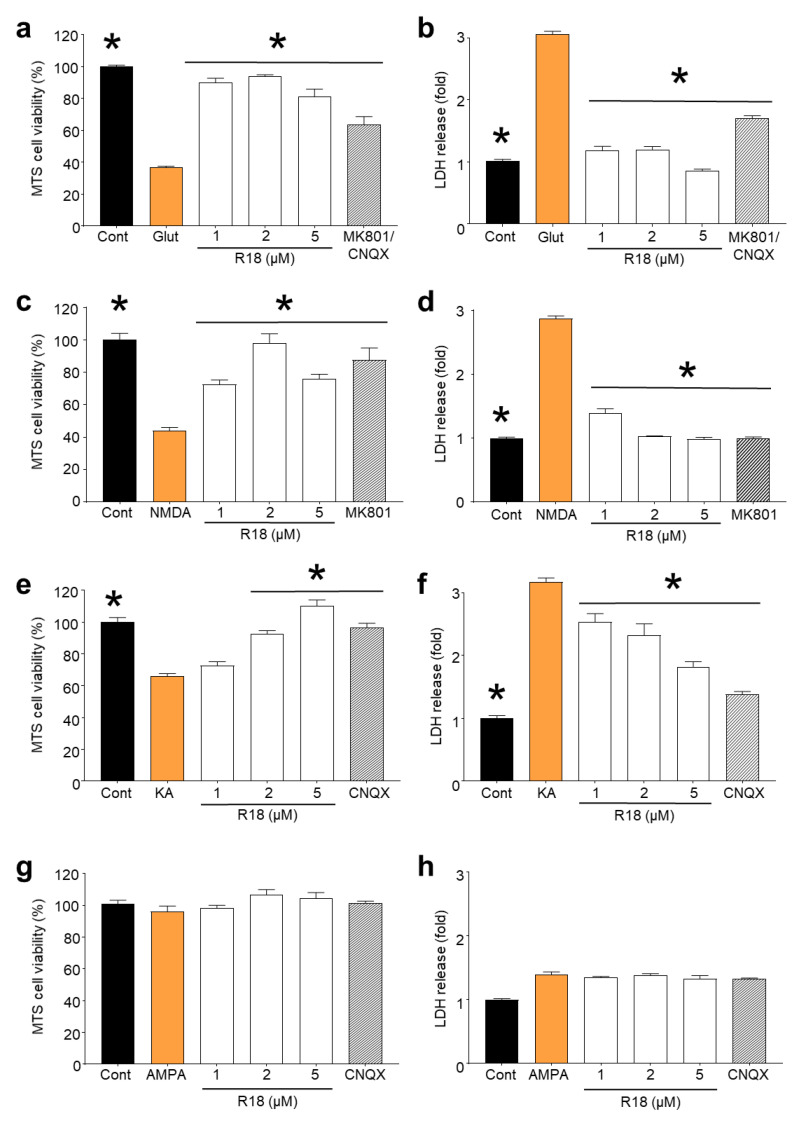
R18 protects primary cortical neurons against excitotoxicity induced by various ionotropic glutamate receptor agonists. Neuronal cultures were pre-treated with R18 (1, 2, and 5 µM) for 10 min and exposed to a 100 µM final concentration of specific iGluR agonists for 5 min. The medium was then replaced, and neuronal viability and death were measured at 24 h post-insult via MTS and LDH assays, respectively. Receptor agonists included (**a**,**b**) glutamate, (**c**,**d**) NMDA, (**e**,**f**) kainic acid, and (**g**,**h**) AMPA. The receptor antagonists, MK801 (10 µM) and CNQX (10 µM), were used as positive controls. Absorbance values were adjusted to represent cell viability (untreated control as 100%). Values are means ± SEM; *n* = 3, with a minimum of eight biological replicates per sample in each experiment; * *p* < 0.05. Experiments were conducted at least three times with independent neuronal cultures.

Dose–response efficacy studies with R18 (1, 2 and 5 µM) following exposure of cortical neurons to different glutamate receptor agonists revealed that the peptide significantly reduced neuronal cell death (*p* < 0.0001) following exposure to glutamic acid, NMDA and KA (Figure 2A–C). R18 provided almost complete protection at the 1 and/or 2 µM concentrations following exposure to glutamic acid and NMDA, and at the 5 µM concentration following exposure to KA. The exposure of cortical neurons to AMPA (100 µM) did not result in a significant level of neuronal death (*p* = 0.7622) (Figure 2D), and therefore the efficacy of R18 to reduce injury could not be determined.

### 2.2. R18 Attenuates Excitotoxic Calcium Influx Stimulated by Ionotropic Glutamate Receptor Agonists

**Figure 3:**
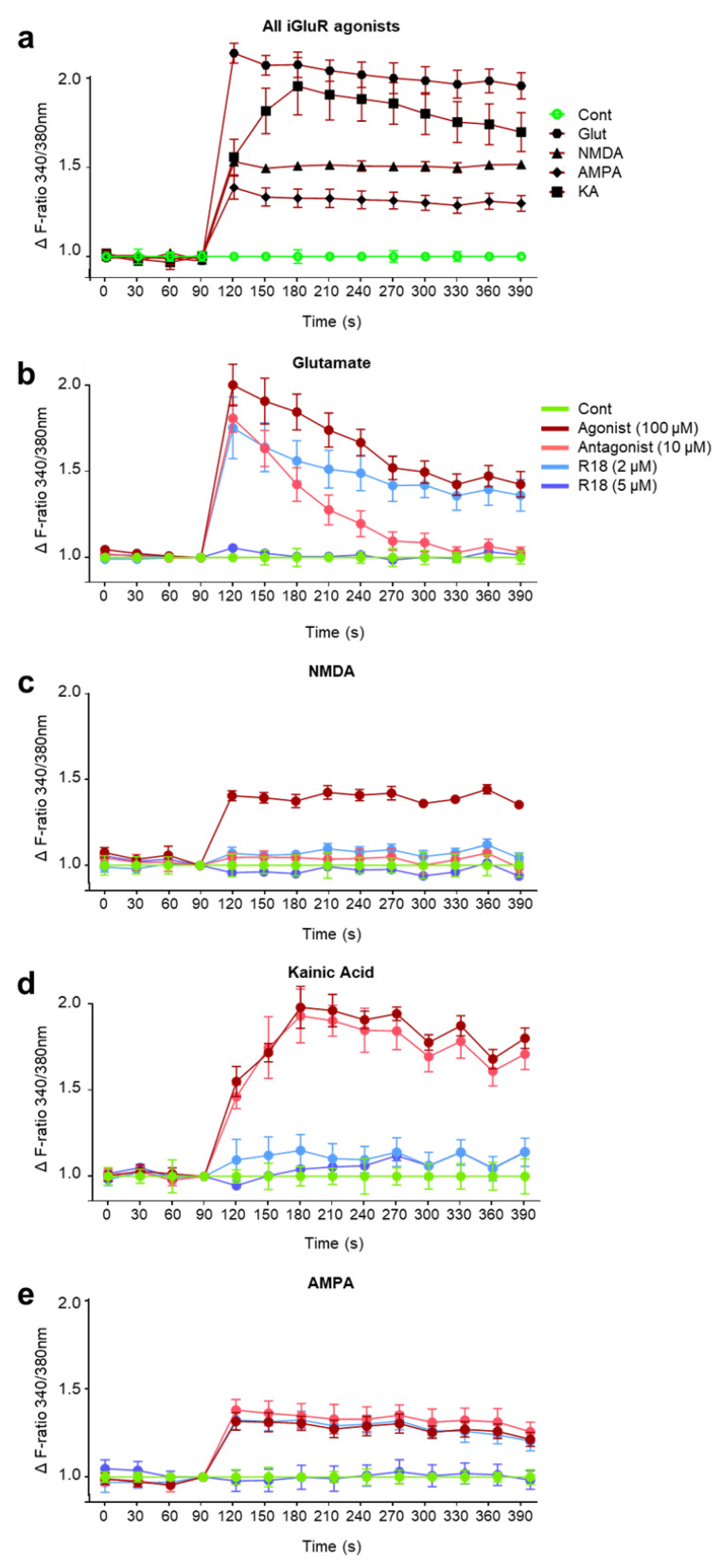
R18 protects primary cortical neurons against iGluR-mediated calcium influx. Cortical neurons were loaded with Fura-2 AM (5 µM in MEM/B27) for 30 min at 37 °C, and the medium was replaced with 50 µL phenol-free Hank’s Balanced Salt Solution (HBSS). Baseline calcium levels were measured every 30 s for 1.5 min prior to the addition of (**a**) iGluR agonists, glutamate, NMDA, AMPA, and KA (100 µM final concentration). Subsequent calcium measurements were made every 30 s for 5 min. To assess the neuroprotective ability of R18 against receptor-mediated calcium influx, neurons were treated with R18 (2 and 5 µM) or receptor blockers (MK801 and/or CNQX; 10 µM) for 10 min prior to stimulation with individual iGluR agonists, (**b**) glutamate, (**c**) NMDA, (**d**) kainic acid, (**e**) or AMPA, and calcium levels were collected every 30 s for a further 5 min. Values are means ± SD; *n* = 3, with a minimum of four biological replicates per sample in each experiment; * *p* < 0.05. Experiments were conducted at least three times with independent neuronal cultures. Fluorescent readings were adjusted to remove background signal and displayed as ΔF ratio (340/380 nm) relative to the untreated control at each timepoint.

An initial study examined neuronal intracellular calcium influx after exposure to the different iGluR agonists at the 100 µM concentration with use of Fura-2 AM to measure rapid intracellular Ca^2+^ transients. The ratiometric dye can be excited at 340 and 380 nm, representing bound and unbound Fura-2 AM dye, and measured at 540 nm to determine the proportion of total calcium that is intracellularly localised (i.e., influx). Glutamic acid elicited the greatest fold increase in intracellular calcium (~2.2-fold), followed by KA (~2-fold), NMDA (~1.5-fold) and AMPA (~1.3-fold) (Figure 3A). To examine whether R18 neuroprotection against excitotoxicity is associated with attenuated intracellular calcium influx, calcium kinetics studies in neuronal cultures were performed using the peptide at the 2 and 5 μM concentrations and iGluR agonists at the 100 µM concentration.

*Glutamic acid*: R18 at 5 µM completely attenuated intracellular calcium influx. In contrast, R18 at 2 µM only had a slight effect in reducing intracellular calcium influx. Treatment with glutamate receptor inhibitors, MK801/CNQX (10 µM/10 μM), resulted in an initial slight reduction in calcium influx. However, the levels of intracellular calcium decreased to untreated control levels by 3 min after glutamic acid addition (Figure 3B).

*NMDA*: R18 at 2 and 5 µM and MK801 (10 µM) completely attenuated intracellular calcium influx (Figure 3C).

*KA*: R18 at 2 and 5 µM attenuated intracellular calcium influx equal to or slightly above baseline levels (Figure 3D). Interestingly, the AMPA/KA receptor antagonist, CNQX (10 μM), did not appear to attenuate intracellular calcium influx.

*AMPA*: R18 at 5 µM completely attenuated intracellular calcium influx. In contrast, R18 at 2 µM and CNQX (10 μM) only had a slight effect in reducing intracellular calcium influx (Figure 3E). Interestingly, the AMPAR inhibitor, CNQX, did not appear to prevent AMPAR-induced calcium influx.

### 2.3. R18 Preserves Mitochondrial Bioenergetics after Glutamic Acid Exposure

**Figure 4:**
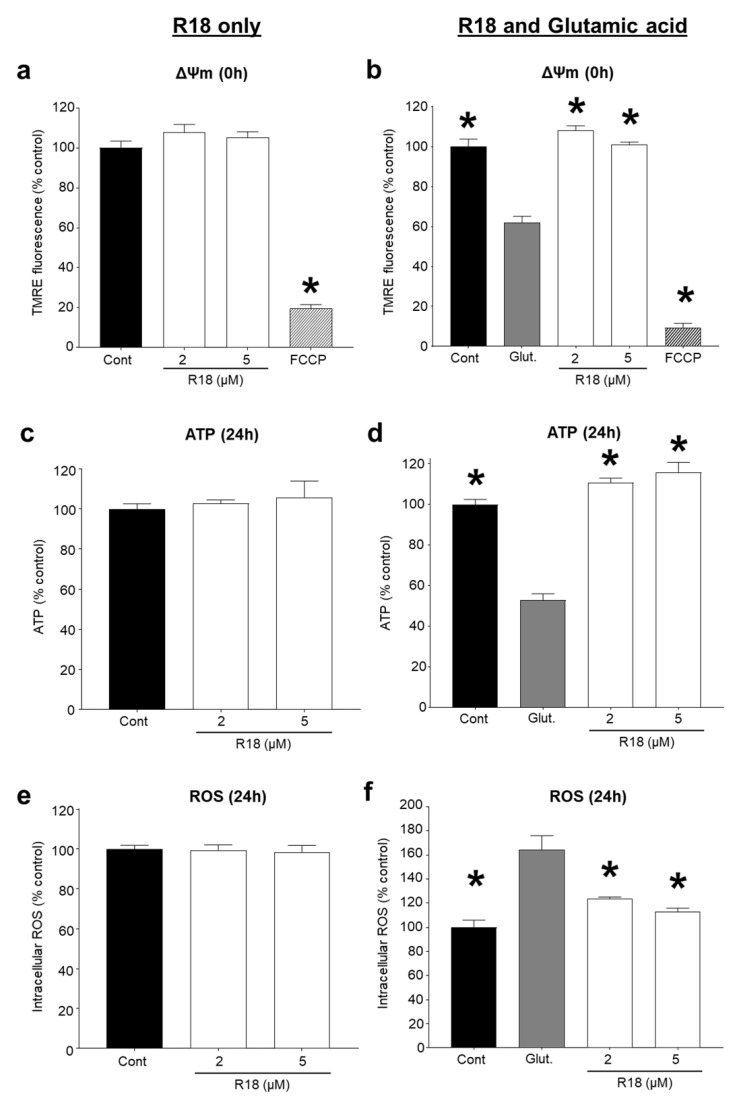
R18 does not disrupt mitochondrial bioenergetics and preserves mitochondrial bioenergetics post-glutamate excitotoxic insult in primary cortical neurons. Cultures received a 5 min glutamate exposure (100 µM final concentration) following a 10 min pre-treatment with R18 (2 or 5 µM). Parameters of mitochondrial bioenergetics were measured fluorometrically post-insult and are represented as fold change in fluorescent intensity. (**a**,**b**) Membrane potential was measured with tetramethylrhodamine ethyl ester (TMRE) immediately post-insult. (**c**,**d**) ATP production and (**e**,**f**) were measured at 24 h post-insult. Background fluorescent values were removed. Background fluorescent values were removed, and experiments were conducted at least three times with independent neuronal cultures. Values are means ± SEM; *n* = 3, with a minimum of eight biological replicates per sample in each experiment; * *p* < 0.05. FCCP (carbonyl cyanide 4-(trifluoromethoxy)phenylhydrazone; 20 µM) was used as a positive control for mitochondrial membrane depolarization.

A central mechanism in excitotoxic neuronal death is mitochondrial calcium overload and dysfunction [31,32]. Therefore, we examined whether R18 exerted any additional abilities to preserve neuronal mitochondrial bioenergetics by measuring mitochondrial membrane potential (∆Ψm), and ATP and ROS levels in neurons treated with R18 alone and exposed to glutamic acid. R18 treatment at 2 and 5 μM did not cause any adverse effects on ∆Ψm, ATP and ROS levels in neurons not exposed to glutamic acid (Figure 4A,C,E). As expected, exposure of neurons to glutamic acid significantly decreased ∆Ψm and ATP levels (*p* < 0.0001), and increased ROS levels (*p* < 0.0001). In neurons exposed to glutamic acid, R18 treatment at 2 and 5 μM significantly preserved ∆Ψm (*p* = 0.0014; *p* = 0.0003) and ATP levels (*p* < 0.0001) and reduced ROS levels (*p* = 0.0021; *p* = 0.0002) (Figure 4B,D,F).

### 2.4. R18 Provides Neuroprotection against Thapsigargin-Induced Injury

**Figure 5:**
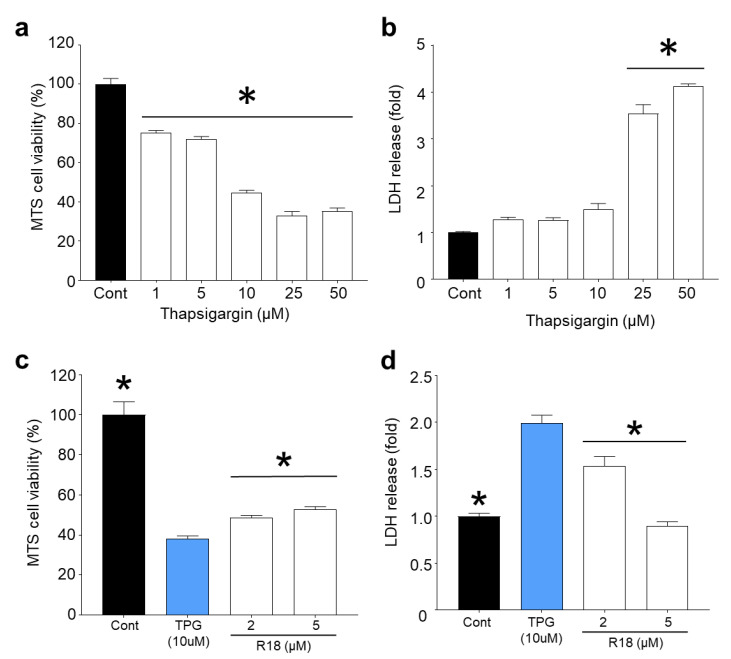
R18 protects primary cortical neurons against thapsigargin-induced neuronal death and ER-mediated calcium release. A 24 h exposure of primary cortical neuronal cultures to thapsigargin (TPG) causes (**a**) a significant reduction in MTS metabolism and (**b**) increased LDH release in a dose-dependent manner. Neuronal cultures were pre-treated with R18 (1, 2, and 5 µM) and exposed to 10 µM final concentration of TPG for 24 h exhibited (**c**) significantly improved MTS metabolism and (**d**) reduced LDH release compared to TPG control at 24 h post-insult. Absorbance values were adjusted to represent cell viability (untreated control as 100%). Values are means ± SEM; *n* = 3, with a minimum of eight biological replicates per sample in each experiment; * *p* < 0.05. Experiments were conducted at least twice with independent neuronal cultures.

Inhibition of the sarco/endoplasmic reticulum calcium ATPase (SERCA) pump causes the gradual increase in cytosolic calcium due to the inability of the ER to sequester calcium, which eventually results in neuronal death [33,34], independent of iGluR-mediated calcium influx. Exposure of cortical neurons to thapsigargin for 24 h resulted in a concentration dependent reduction in cell viability and cell death (Figure 5A,B). Based on MTS and LDH release assays, thapsigargin at the 25 µM (*p* < 0.0001) and 50 μM (*p* < 0.0001) was highly toxic, causing greater than 70% neuronal death. In contrast, lower doses of thapsigargin (1, 5 and 10 μM) resulted in modest toxicity causing between 10–20% cell death. Treatment of neurons with R18 at 2 µM (*p* = 0.0467) and 5 µM (*p* = 0.001) significantly reduced neuronal death following exposure to thapsigargin (10 µM) (Figure 5D). Interestingly, based on LDH release, R18 at 5 µM completely inhibited cell death, whereas MTS metabolism in the same neuronal cultures was significantly reduced (*p* = 0.001), but above the thapsigargin treated control (Figure 5C). This indicates that while R18 was able to preserve neuronal viability, the continued exposure of neurons to thapsigargin and ER stress affected the ability of the neurons to metabolise the MTS substrate.

## 3. Discussion

### 3.1. Protective Effects of R18 against iGluR agonist Induced Excitotoxicity

In the present study, we further characterised the anti-excitotoxic actions of the poly-arginine-18 (R18) peptide in primary cortical neuronal cultures. In doing so, it was established that R18 provides neuroprotection against iGluR-mediated excitotoxicity induced by NMDA and KA receptor overstimulation, which is in line with a previous study in our laboratory examining the anti-excitotoxic actions of the D-enantiomer R9 poly-arginine peptide (R9D) [35]. In addition, we have shown that R18 reduces NMDA-, KA- and AMPA-induced neuronal intracellular calcium influx, which confirms the broad-acting capacity of the peptide to antagonize NMDA, KA and AMPA receptor activation, thereby limiting excitotoxic intracellular calcium entry. Interestingly, the competitive AMPAR/KAR inhibitor, CNQX, did not prevent AMPA- or KA-induced neuronal intracellular calcium influx. At present, the reason for this result is not known, and therefore future studies should examine AMAP- and KA-mediated neuronal calcium influx with CNQX alongside other AMPAR and KAR antagonists, such as perampanel and LY382884. Nonetheless, these findings are supported by an earlier study which demonstrated that short arginine-rich peptides (2–6-mers) can inhibit NMDA and AMPA evoked ionic currents and antagonize the vanilloid receptor 1 (VR1/TRPM1) in Xenopus oocytes expressing NMDA, AMPA, and VR1 receptors, respectively [36,37].

It is of interest that the efficacy of R18 in maintaining neuronal viability at 2 and 5 µM differed with the different ionotropic glutamate receptor agonists (Figure 2). For example, R18 at 5 µM appeared less effective than at 2 µM at inhibiting glutamic acid- and NMDA-mediated excitotoxicity, while following kainic acid exposure R18 followed a dose–response effect. The reduced efficacy of R18 at the higher 5 µM concentration has been observed previously following glutamic acid excitotoxicity and viability assessment using the MTS assay [35], and is likely due to the peptide exerting an adverse effect on neuronal metabolism, rather than cell death, as LDH release levels were not elevated in the 5 µM treatment group compared to the 2 µM treatment group. One explanation is that pre-treatment of neuronal cultures with R18 at high concentrations causes elevated intracellular levels of the peptide, which when combined with NMDA receptor activation adversely affects mitochondrial function, as CARPs are known to target this organelle. Alternatively, R18 may be causing an excessive and prolonged inhibition or downregulation of neuronal surface receptors such as NMDARs or neurotrophic receptors that are important for maintaining neuronal biochemical function.

A potential mechanism whereby R18 has the capacity to antagonize the function of neuronal cell surface ion channel receptors is by inducing the endocytic internalisation of the receptors and thereby reducing excitotoxic calcium influx [18]. For example, we have previously demonstrated that in cultured cortical neurons the poly-arginine-12 (R12) peptide reduces neuronal cell surface levels of the NMDA receptor subunit protein, NR2B [29]. In addition, other CARPs have also been demonstrated to reduce neuronal cell surface expression of NMDA receptors and of other ion channel receptors including TRPV1, NCX, CaV2.2, CaV3.3 [38,39,40,41,42,43,44]. Whether R18 and other CARPs also reduce neuronal cell surface levels of AMPA and KA receptors will require further investigation. However, it is noteworthy that the arginine-rich cell-penetrating peptides R9, TAT, and penetratin induce internalisation of tumor necrosis factor and epidermal growth factor receptors in HeLa cells [43]. An alternative, but not mutually exclusive mechanism whereby R18 may antagonize ion channel receptor activity, is through electrostatic interactions with negatively charged amino acids located in or near the entrance to the pore of the receptor. For example, positively charged guanidino moieties, which are present in arginine residues and related molecules, such as agmatine, are capable of interacting with and affecting the function of NMDA receptors and voltage-gated calcium channels [45,46]. Therefore, further studies are required to determine the capacity of R18 to reduce neuronal surface levels of iGluRs and other ion channel receptors, and to inhibit receptor function directly by an electrostatic mechanism.

### 3.2. Protective Effects of R18 on Mitochondrial Function

Another important finding of this study is that R18 has the capacity to reduce glutamate excitotoxicity induced mitochondrial dysfunction by maintaining Δψm and ATP production, and to reduce ROS generation. Previous studies have shown that CARPs are able to preserve mitochondrial function and integrity through both direct and indirect means. For example, the positively charged arginine residue containing Szeto–Schiller (SS) tetramers (e.g., SS-20, SS-31; net charge + 3) have been shown to enter mitochondria and can prevent release of cytochrome c from the mitochondrial intermembrane space, maintain mitochondrial bioenergetics, and reduce ROS production [47,48,49,50]. In addition, in isolated mitochondrial preparations, poly-arginine-4 (R4; net charge + 4) has been shown to prevent opening of the mitochondrial permeability pore (mPTP) induced by high calcium concentrations, which represents a critical mitochondrial-regulated signal for irreversible cell death [28]. Moreover, the ability to prevent mPTP opening is charge-dependent and therefore it is likely that longer poly-arginine peptides (e.g., R18; net charge + 18) are also able to target and inhibit mPTP opening. In addition, CARPs have exhibited innate antioxidant properties [18], due at least in part to the presence of arginine residues, which may contribute to their ability to attenuate the damaging effects of ROS production associated with excitotoxicity. For example, the ROS-scavenging abilities of arginine and other guanidinium-containing compounds, including guanidine, aminoguanidine, methylguanidine and creatine, have been demonstrated with superoxide hydrogen peroxide and peroxynitrite, and neurotoxic reactive aldehyde lipid peroxidation by-products, such as 4-hydroxynonenal and malondialdehyde [51,52,53,54,55,56,57]. However, further studies are required to determine whether the protective effects of R18 are mediated directly as a result of localisation of the peptide to mitochondria, and/or through an indirect mechanism.

### 3.3. Protective Effects of R18 against Thapsigargin Neurotoxicity

While we have confirmed that R18 has potent neuroprotective actions in glutamate receptor-mediated excitotoxicity, our findings indicate that it also has the capacity to reduce neurotoxicity and prevent neuronal cell death in a thapsigargin neuronal injury model in which intracellular calcium levels are raised independent of glutamate receptor-mediated calcium influx [34]. Although the exact mechanism whereby R18 protects neurons from thapsigargin-induced neuronal death is yet to be elucidated, it is possible that the peptide helps to maintain mitochondrial integrity following exposure to elevated intracellular calcium concentrations. To this end, it appears that while R18 was highly neuroprotective at the 2 µM concentration in the excitotoxicity injury models, this concentration of the peptide in the calcium kinetic studies did not always result in high-level inhibition of intracellular calcium influx, suggesting that there were additional factors (i.e., mitochondrial preservation) that contributed to this overall neuroprotective benefit.

### 3.4. Potential Future Clinical Application of R18

R18 represents a potential broad-acting neuroprotective agent for the treatment of both acute and chronic neurological disorders. In acute clinical settings such as stroke and traumatic brain injury, ideally R18 should be administered intravenously to provide fast delivery to the brain. In chronic clinical settings such as Parkinson’s disease and Alzheimer’s disease, the peptide could be delivered as a nasal spray, or alternatively the d-enantiomer version of R18, which is likely to be resistant to proteolytic degradation could be administered orally, as has been proposed for RD2, a d-isomer CARP being developed for Alzheimer’s disease [58].

## 4. Materials and Methods

### 4.1. Peptides

Poly-arginine-18 (R18; H-RRRRRRRRRRRRRRRRRR-OH) peptide was synthesized by Mimotopes (Mulgrave, VIC, Australia) and purified to 98% by high-performance liquid chromatography. Peptides were prepared as 500 µM stocks in water for irrigation (Baxter, NSW, Australia) and stored at −20 °C prior to use.

### 4.2. Primary Cortical Neuronal Cultures

Briefly, cortical neuronal tissue was extracted from E18 Sprague–Dawley rat embryos, dissociated, resuspended in Neurobasal/2% B27 supplement (B27) and seeded at approximately 55,000 cells/well into clear (Nunc, Scoresby, VIC, Australia) or black (Costar) 96-well plates, pre-coated with poly-lysine (Sigma-Aldrich, Castle Hill, NSW, Australi). Plates were maintained in a CO_2_ incubator (37 °C, 95% air balance, 98% humidity, 5% CO_2_) until use on day in vitro 10, cultures routinely comprise > 97% neurons and 1–3% astrocytes. Approval for the use of E18 Sprague–Dawley rat embryos for isolation of cortical tissue was obtained by the University of Western Australia Animal Ethics Committee (RA/3/100/1432).

### 4.3. iGluR agonist Excitotoxicity Model

Neuronal excitotoxic iGluR activation was achieved by treating cortical neurons with iGluR agonist glutamic acid (l-glutamic acid; Sigma-Aldrich, #PHR1107), N-methyl-D-aspartate (NMDA; Sigma-Aldrich, #M3262), α-amino-3-hydroxy-5-methyl-4-isoxazolepropionic acid (AMPA; Sigma-Aldrich, #A6816), or kainic acid (KA; Sigma-Aldrich, #K2389). Preparation of primary cortical neuronal cultures was conducted as previously described [59]. R18 was added to cortical neuronal cultures 10 min prior to exposure to individual iGluR agonists by removing the medium and adding 50 µL of Minimal Essential Medium (MEM)/2% B27 containing peptide (2 µM). To induce excitotoxicity, 50 µL of MEM/2% B27 containing the agonist (200 µM; final concentration 100 µM) was added to the culture wells and incubated in a CO_2_ incubator for 5 min (note: peptide concentration reduced to 1 µM during this step). Following the 5 min exposure, the medium was replaced with 100 µL of MEM/2% B27 and cultures were incubated for a further 24 h in a CO_2_ incubator. Untreated controls with or without receptor agonist treatment underwent the same incubation steps and medium additions.

### 4.4. Thapsigargin Intracellular Calcium Injury Model

To induce raised intracellular calcium levels in neurons in a receptor-independent manner, the irreversible SERCA pump inhibitor, thapsigargin (Sigma-Aldrich, #T9033), was used. Briefly, R18 was added to cortical neuronal by removing media, adding 50 µL of MEM/2% B27 containing peptide (2 µM) before incubating for 10 min incubation in a CO_2_ incubator. After the 10 min incubation 50 µL of MEM/2% B27 containing thapsigargin (prepared as 2x stock; 10 µM final concentration) was added to the wells and cultures incubated in a CO_2_ incubator for 24 h. Untreated controls with or without thapsigargin treatment underwent the same incubation steps and medium additions.

### 4.5. MTS Cellular Viability and LDH Cytotoxicity Assays

At 24 h post-injury, cell viability was assessed quantitatively using the CellTiter 96 Aqueous Cell Proliferation MTS assay (Promega, Hawthorn, VIC, Australia; #G3582) and the CytoTox 96^®^ Non-Radioactive Cytotoxicity LDH release assay (Promega, #G1780) as per the manufacturer’s instructions. The MTS assay determines cell viability by assessing the metabolic capacity of cells to reduce a tetrazolium salt (MTS), which is measured spectrophotometrically at 490 nm.

The LDH release assay measures lactate dehydrogenase activity (LDH) released from dead cells in an enzymatic reaction involving the conversion of a tetrazolium salt (INT) into a red formazan product that is measured spectrophotometrically at 490 nm. Cell death was presented as fold change of the untreated control, after removal of background signal.

### 4.6. Intracellular Calcium Kinetics

Measurement of iGluR-induced intracellular calcium influx [Ca^2+^]_i_ was performed using the fluorogenic Fura-2 AM dye, as previously described [30]. Briefly, cortical neuronal cultures were loaded with the Fura-2 AM dye (5 µM in 50 µL MEM/2% B27 per well) and incubated at 37 °C for 30 min in the dark. R18 (2 and 5 µM) or iGluR antagonists, MK801 (Sigma-Aldrich, #M107) and CNQX (Sigma-Aldrich, #C127), were applied for 10 min in MEM/2% B27. The cells were rinsed with 100 µL of Hank’s Balanced Salt Solution (HBSS, pH 7; Life Technologies, Mulgrave, VIC, Australia), and baseline intracellular Fura-2 AM calcium measurements recorded (Em/Ex = 340 nm and 380 nm/510 nm) for 3 min. Cells were then exposed to the individual iGluR agonists, glutamic acid, NMDA, AMPA, or KA (100 µM final concentrations in HBSS) and Fura-2 AM calcium measurements recorded for a further 10 min. Untreated control wells received the same medium additions, without peptide treatment or iGluR agonists. Background signal (no Fura-2 AM dye) was subtracted for each wavelength at each timepoint, and data were adjusted to reflect the ΔF ratio (340/380 nm), relative to untreated control wells.

### 4.7. ATP Measurement in Cortical Neuronal Cultures

ATP levels in neuronal cultures were measured 24 h after exposure to glutamic acid using the Luminescent ATP Detection Assay Kit (Abcam, Cambridge, MA, USA, #ab113849) according to the manufacturer’s protocol. Cortical neurons seeded in black 96-well plates were treated with R18 (2 and 5 µM) by removing the medium in wells and adding 50 µL of MEM/2% B27 containing peptide before incubating in a CO_2_ incubator for 10 min. Fifty microliters of the proprietary cell lysis buffer was added, and the plate was incubated for a further 5 min on an orbital shaker (700 rpm), before the addition of 50 µL of substrate to wells and a further 5 min incubation on the orbital shaker. Plates were subsequently measured on a microplate luminometer (Cytation5, BioTek, Winooski, VT, USA). ATP values represent percentage change compared with untreated controls (taken as 100%) after subtraction of background values from each treatment (the medium only). Experiments were performed in triplicate.

### 4.8. Reactive Oxidative Species (ROS) Detection

ROS levels in neuronal cultures were measured 24 h after exposure to glutamic acid using the 2′,7′-dichlorofluorescin diacetate (DCFDA/H_2_DCFDA) fluorescent probe (Abcam, #ab113851), which is a cell-permeable dye that is cleaved by cellular esterases and subsequently oxidized by intracellular ROS, including hydroxyl and peroxyl species. Oxidized DCFDA forms the fluorescent dichlorofluorescein (DCF) compound that is detected by fluorescent spectroscopy (Ex/Em = 495 nm/529 nm). Fluorescent DCF values represent the percentage change compared with untreated control (taken as 100%) after subtraction of background signal (the medium only). Experiments were performed in triplicate.

### 4.9. Mitochondrial Membrane Potential (ΔΨm)

Mitochondrial membrane potential (ΔΨm) was assessed in neuronal cultures immediately after glutamic acid exposure using the TMRE-Mitochondrial Membrane Potential Assay Kit (Abcam, #ab113852). Tetramethylrhodamine ethyl ester (TMRE) is a cell-permeable dye that accumulates in active mitochondria resulting in an increase fluorescent intensity, which can be measured fluorometrically. Briefly, after the removal of the medium in wells containing glutamic acid, 50 µL of MEM/2% B27 containing TMRE (1 µM) was added to neuronal cultures and incubated in a CO_2_ incubator for 20 min. Cells were washed with 100 µL of HBSS, and TRME fluorescence measured using the Cytation5 plate reader (Ex/Em = 549 nm/575 nm). As a positive control, to disrupt the ΔΨm neuronal cultures were exposed to the electron transport chain (ETC) uncoupler, FCCP (carbonyl cyanide 4-(trifluoromethoxy)phenylhydrazone; 20 µM) for 10 min prior to TMRE fluorescence measurement. TMRE fluorescence values represented the percentage change in fluorescence relative to untreated control (taken as 100%) after subtraction of background measurements (the medium only).

### 4.10. Statistical Analysis

Statistical analysis was performed using Prism statistical software (Version 8.02, San Diego, CA, USA). For data relating to viability and biochemical assays, experimental group differences were analyzed using a one-way analysis of variance (ANOVA) and subsequent Dunnett post-hoc tests. Data are presented as the mean ± SEM, with *p* < 0.05 considered statistically significant. Experiments were repeated at least twice independently, with a minimum of eight biological replicates for cell viability and cell death assays, or a minimum of four biological replicates for biochemical assays.

## 5. Conclusions

Together, the findings of this study indicate that glutamate receptor modulation as well as preservation of mitochondrial bioenergetics represent key neuroprotective actions of poly-arginine peptides in glutamate excitotoxicity. Such findings therefore support the potential utility and further investigation of this group of peptides as neuroprotective agents for acute forms of brain injury such as ischaemic stroke and traumatic brain injury, as well as chronic neurodegenerative disorders, including ALS, AD and PD in which glutamate excitotoxicity [60], oxidative stress [61], and mitochondrial dysfunction [11] are considered to be key players in disease pathogenesis.

## 6. Patents

B.P. Meloni and N.W. Knuckey are named inventors of several patent applications (Provisional Patents: 2013904197; 30/10/2013 and 2014902319; 17/6/2014 and PCT/ AU2014/050326; 30/10/2104) regarding the use of arginine-rich peptides as neuroprotective agents.
